# Blocking the CXCL1-CXCR2 axis enhances the effects of doxorubicin in HCC by remodelling the tumour microenvironment via the NF-κB/IL-1*β*/CXCL1 signalling pathway

**DOI:** 10.1038/s41420-023-01424-y

**Published:** 2023-04-10

**Authors:** Huiyong Zhao, Sheng Wei, Dachen Zhou, Yongfan Liu, Zicheng Guo, Chuibao Fang, Xiaoxi Pang, Fei Li, Hui Hou, Xiao Cui

**Affiliations:** 1 Department of General Surgery, The Second Hospital of Anhui Medical University, Hefei, China; 2 Department of Nuclear Medicine, The Second Hospital of Anhui Medical University, Hefei, China

**Keywords:** Cancer microenvironment, Hepatocellular carcinoma

## Abstract

Inflammation is a core mechanism for oncogenesis. Chemokines act as important mediators of chronic inflammation and the tumour inflammatory response. However, there is limited information on chemokines in hepatocellular carcinoma (HCC), a disease for which almost all cases are derived from chronic liver inflammation. Here, we explored the protumor effects of CXCL1, a commonly elevated inflammatory chemokine in cirrhosis, in HCC. The protumor role was confirmed in clinical samples from HCC patients. CXCL1 enhanced tumorigenesis in the hepatic inflammatory microenvironment directly by acting on tumour cells and indirectly through promoting the recruitment of macrophages. The increase in the number of macrophages in the tumour microenvironment (TME) promoted tumour cell epithelial-mesenchymal transition (EMT) and significantly increased CXCL1 levels in the TME partly through NF-κB/IL-1*β* activation. To investigate the potential therapeutic value of CXCL1 in HCC with an inflammatory background, an antibody blocking CXCL1 was used alone or combined with the chemotherapy agent doxorubicin (DOX), with the goal of reshaping the TME. It has been shown that blocking CXCL1-CXCR2 inhibits tumour progression and reduces macrophage recruitment in the TME. The combination regimen has been shown to synergistically reduce the number of pro-tumour macrophages in the TME and suppress tumour progression. This provides insight into therapeutic strategies for treating HCC patients with high CXCL1 expression.

## Introduction

HCC is one of the most lethal malignant tumours [1]. Although rapid advancements in methods and strategies for HCC have been made during the past several years, the long-term prognosis of patients is still far from satisfactory [1, 2]. In terms of aetiology, HCC is often attributed to a sustained chronic inflammatory state in the liver caused by viral or nonviral hepatitis [3, 4]. A series of research results showed that in the process of occurrence and development of HCC, an imbalance in inflammatory cells induces cirrhosis progression, and the involved inflammatory cells show pro-tumour functions [5, 6] by regulating tumour growth, invasion, and vascular invasion in the tumour microenvironment [7].

CXCL1, a chemokine that acts as an important proinflammatory mediator by driving the migration of a variety of immune cells in cirrhosis has been fully explored and found to specifically bind to CXCR2 [8]. Previous studies have revealed that CXCL1 and its receptor CXCR2 are involved in the angiogenesis, tumorigenesis and metastasis of different types of tumours, such as rectal cancer, breast cancer and HCC. CXCL1 is capable of activating inflammasomes by stimulating the release of IL-1*β* through the G-protein-coupled receptor CXCR2 [9]. Kupffer cells/monocytes have been widely regarded as the main source of CXCL1 in liver inflammation [10, 11]. High expression of CXCL1 can predict the recurrence of HCC in patients and promote tumour progression by increasing tumour cell metabolism and epithelial mesenchymal transformation (EMT) [12, 13]. The effect of inhibiting CXCL1 in HCC with an inflammatory background is unclear [11]. Here, we investigated the prognostic value of CXCL1, and the relationship between CXCL1 expression and macrophages infiltrations in HCC clinical samples through analysis of a database and a cohort from a single center. Negative regulation of the immune response mediated by CXCL1 involving tumour cells and macrophages in the microenvironment of HCC has been investigated in vivo and in vitro. Chemotherapy has been considered an effective way to treat tumours; however, chemotherapy resistance is still a complex clinical problem [12]. Moreover, the CXCL1/CXCR2 axis is still thought to be closely related to tumour resistance.

In this study, we explored the clinical value of CXCL1 in HCC and investigated the ability of anti-CXCR2 antibody alone or combined with doxorubicin (DOX) to produce synergistic antitumour effects in HCC.

## Results

### High CXCL1 expression predicts a poor prognosis in HCC and is positively related to macrophage enrichment

**Fig. 1:**
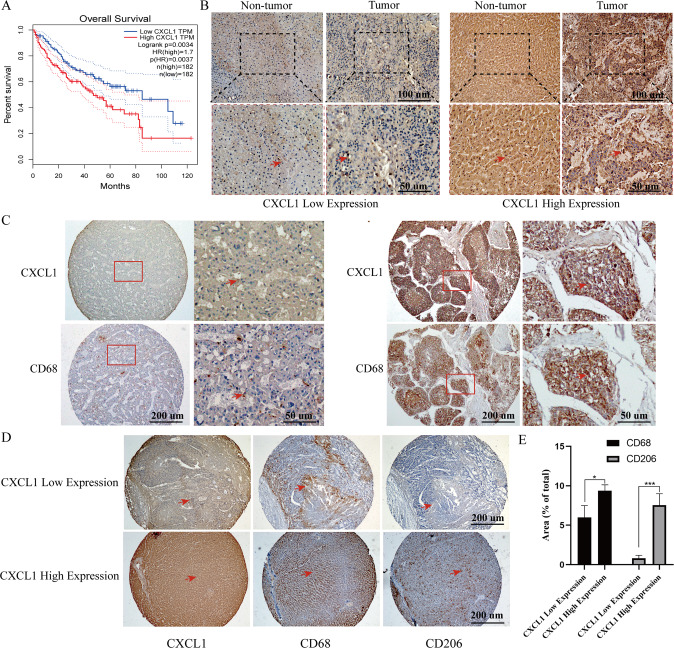
High CXCL1 expression predicts a poor prognosis in HCC, and is positively related to macrophage enrichment. **A** Overall survival plot of CXCL1 expression in HCC patients was analysed through the GEPIA database (*n* = 364). **B** The expression of CXCL1 in tumour tissues and adjacent nontumor tissues was measured by IHC. Arrows indicate positive staining. Scale bar, 100 μm (up) and 50 μm (down). **C**–**E** CD68 and CXCL1 staining and representative images of the same tissue. **D** CXCL1, CD68, and CD206 staining in the same tissues. Arrows indicate positive staining. Scale bar, 200 μm. **E** The relationship between CXCL1 expression and the CD206^+^/CD68^+^ cell ratio. Data are expressed as the mean ± SEM. **P < 0.01, ns, nonsignificant (*n* = 5/group).

CXCL1 expression levels in tumour samples from HCC patients were analysed through the GEPIA database. Patients with high CXCL1 expression had a poor prognosis (Fig. 1A). To further confirm the relationship between prognosis and the pathological characteristics of HCC patients with various expression levels of CXCL1, we performed immunohistochemistry of HCC and paired adjacent nontumorous tissues on paraffin-embedded TMAs. The results showed that CXCL1 expression levels were similar between HCC tissues and paired adjacent nontumorous tissues (Fig. 1B). The expression level of CXCL1 was higher in most HCC patient tissue samples than in corresponding normal samples and was positively related to cirrhosis and tumour microvascular invasion (Table 1). A positive relationship between cirrhosis and CXCL1 levels was also found in the analysis paired adjacent tissues (Table S2). We used immunohistochemistry to further assess the relationship between CXCL1 and macrophages in HCC tissues. Notably, the proportion of CD68-positive cells was positively related to CXCL1 expression (Fig. 1C). We observed the enrichment levels of the total macrophage marker CD68 and M2-type macrophage marker CD206 in tissues with different CXCL1 expression levels. M2 macrophages can facilitate tumour cell growth, invasion, and metastasis and induce chemoresistance by secreting chemokines and cytokines. HCC tissues with high CXCL1 expression contained more CD206-positive cells than HCC tissues with low CXCL1 expression (Fig. 1D, E).

**Table 1:** Correlation between CXCL1 expression in tumour tissues from HCC patients and corresponding clinicopathologic parameters.

Factors	CXCL1 expression		
	Low(*n* = 30)	High(*n* = 21)	*P*-value
Sex (male/female)	28/2	19/2	1.000
Age (≤ 65/> 65 years)	24/16	17/4	1.000
AFP (low/high)	14/16	9/12	0.788
Cirrhosis (yes/no)	19/11	20/1	0.021*
Microvascular invasion (yes/no)	7/23	11/10	0.033*

*P < 0.05.

### CXCL1 promotes proliferation and metastasis and affects EMT in HCC cells

**Fig. 2:**
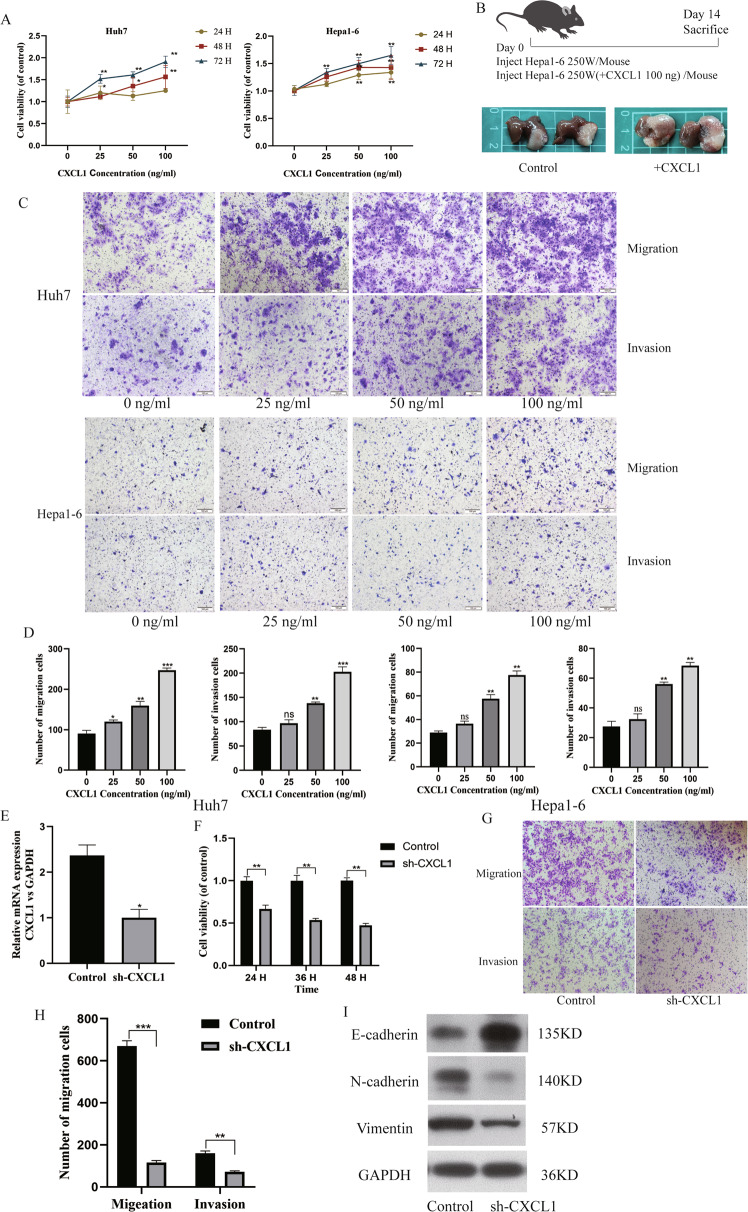
Potential role of CXCL1 in HCC progression. **A** Different concentrations of CXCL1 recombinant protein (0 ng, 25 ng, 50 ng, 100 ng/ml) promoted cell proliferation in Huh7 and Hepa1–6 cells, as demonstrated by MTT assay at different times (24 h, 48 h, 72 h). Data are expressed as the mean ± SEM. *P < 0.05, **P < 0.01 (*n* = 6/group). **B** Schematic representation of injected cells and photograph of primary tumours from different groups (*n* = 5/group). **C**, **D** Photographs and quantification of Transwell assays results indicated that the addition of recombinant CXCL1 protein increased the metastatic capability of HCC cells. **E** qRT-PCR analysis was conducted to detect the CXCL1 levels in Huh7 cells that were stably transfected with shRNA vectors or a control vector. Data are expressed as the mean ± SEM. *P < 0.05 (*n* = 3/group). **F** MTT assay revealed that the proliferation of sh-CXCL1-Huh7 cells was decreased. Data are expressed as the mean ± SEM. **P < 0.01 (*n* = 6/group). **G**, **H** Photograph and quantification of sh-CXCL1-Huh7 and control-Huh7 cells in the Transwell assay to detect metastasis capability ***P < 0.001. **I** Western blot analysis showed that sh-CXCL1 inhibited EMT signalling.

In MTT assays, compared to that in the control group, the proliferation of HCC cells was promoted when different concentrations of CXCL1 recombinant protein were added, and the proportion of proliferating cell was positively correlated with the concentration of CXCL1 (Fig. 2A). Next, we demonstrated in mice that transfusions of Hepa1–6 cells stimulated with CXCL1 recombinant protein produced larger tumours in the liver than unstimulated cells (Fig. 2B). It was also observed that the number of HCC cells that migrated through the Transwell membrane with or without Matrigel coating increased when exogenous CXCL1 recombinant protein was added (Fig. 2C, D). We used Huh7 cells stably transfected with an empty vector (Control) or CXCL1 silencing vector (sh-CXCL1) to further verify our results. To observe the level of CXCL1 downregulation, PCR was used for quantification. (Fig. 2E). In contrast to the effects of recombinant CXCL1 treatment, CXCL1-downregulation slowed tumour growth (Fig. 2F) and decreased the number of cells that migrated through the membrane with or without Matrigel coating (Fig. 2G, H). CXCL1 significantly affects the epithelial-mesenchymal transition (EMT) pathway. The EMT marker protein E-cadherin was upregulated, and N-cadherin and Vimentin were downregulated when CXCL1 expression was decreased (Fig. 2I).

### Tumour-associated macrophages (TAMs) stimulate the secretion of CXCL1 to induce M2 macrophages polarization and affect the migration and invasion of HCC cells

**Fig. 3:**
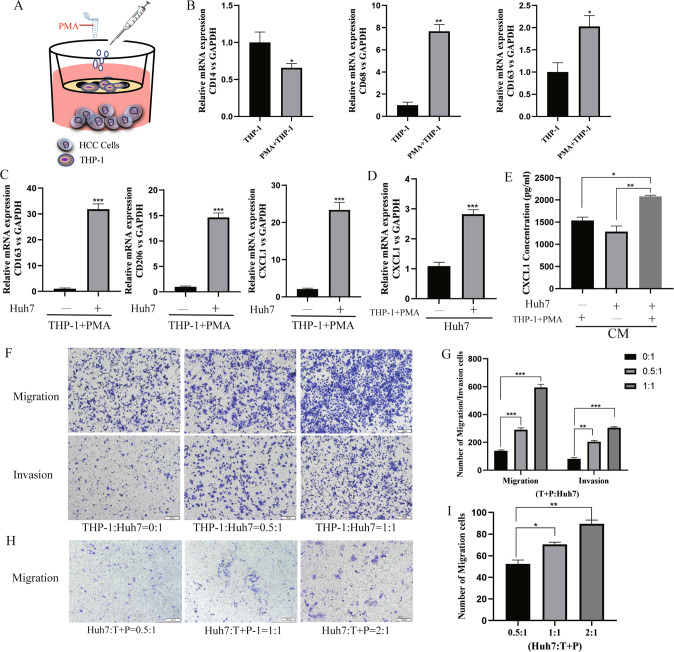
Coculture of PMA-primed THP-1 cells with Huh7 cells promoted M2 polarization, macrophage CXCL1 secretion and HCC cell migration and invasions. **A** Schematic representation of the in vitro model for the coculture of PMA-primed THP-1 cells with Huh7 cells. **B** CD14, CD68, and CD163 production detected in PMA-primed THP-1 cells by qRT-PCR. Data are expressed as the mean ± SEM. *P < 0.05, **P < 0.01 (*n* = 3/group). **C**, **D** The relative mRNA expression of CD163, CD206, and CXCL1 in macrophages in the coculture system. Data are expressed as the mean ± SEM. ***P < 0.001 (*n* = 3/group). **E** CXCL1 levels in the coculture supernatant (CM) were measured by ELISA. Data are expressed as the mean ± SEM. *P < 0.05, **P < 0.01 (*n* = 5/group). **F**, **G** Photograph and quantification of Huh7 cell migration and invasion in the coculture system at different ratios of macrophages to Huh7 cells. **H**, **I** Photograph and quantification of macrophage migration in the coculture system at different ratios of macrophages to Huh7 cells.

Human monocytes (THP-1) cells were induced to differentiate into macrophages by PMA and cocultured with HCC cells, as previously described in the methods (Fig. 3A). We observed that THP-1 cell morphology shifted from round to irregularly shaped after PMA induction, indicating a change from suspension cells to adherent cells (Fig. S1A). The induction of THP-1 cells into macrophages was confirmed by PCR. The expression of CD68 and CD163 was increased, while the expression of CD14, a marker of monocytes, was decreased compared with that in THP-1 cells (Fig. 3B). In this coculture system, we called the macrophage TAMs, and the Huh7 cells were called co-Huh7 cells. Typical markers of M2 macrophages (CD163 and CD206) were upregulated in TAMs, and CXCL1 was also upregulated (Fig. 3C). The same result was also revealed in HCC cells and conditioned medium (CM) (Fig. 3D, E). In the human HCC sample data from the GEPIA database, it was found that CXCL1 expression was higher in M2-like macrophages than in macrophages with other phenotypes (Fig. S1B). Based on the above results, we predicted that coculture of macrophages and HCC cells can stimulate the secretion of CXCL1 in the TME and induce macrophages polarization toward to M2-phenotype. To explore the effect of M2 macrophages in inducing EMT and accelerating migration and invasion behaviour, we performed a Transwell assay. With the increasing proportion of macrophages in the coculture system, the number of HCC cells migrating through the chamber membrane increased accordingly (Figs. 3F, G, S1C, D). It was also observed that the migration of macrophages was enhanced when the ratio of HCC cells increased in the coculture microenvironment (Figs. 3H, I; S1E, F).

### Blocking the CXCL1-CXCR2 axis with anti-CXCR2 antibody reduces the secretion of CXCL1 and inhibits the proliferation and metastasis of HCC

**Fig. 4:**
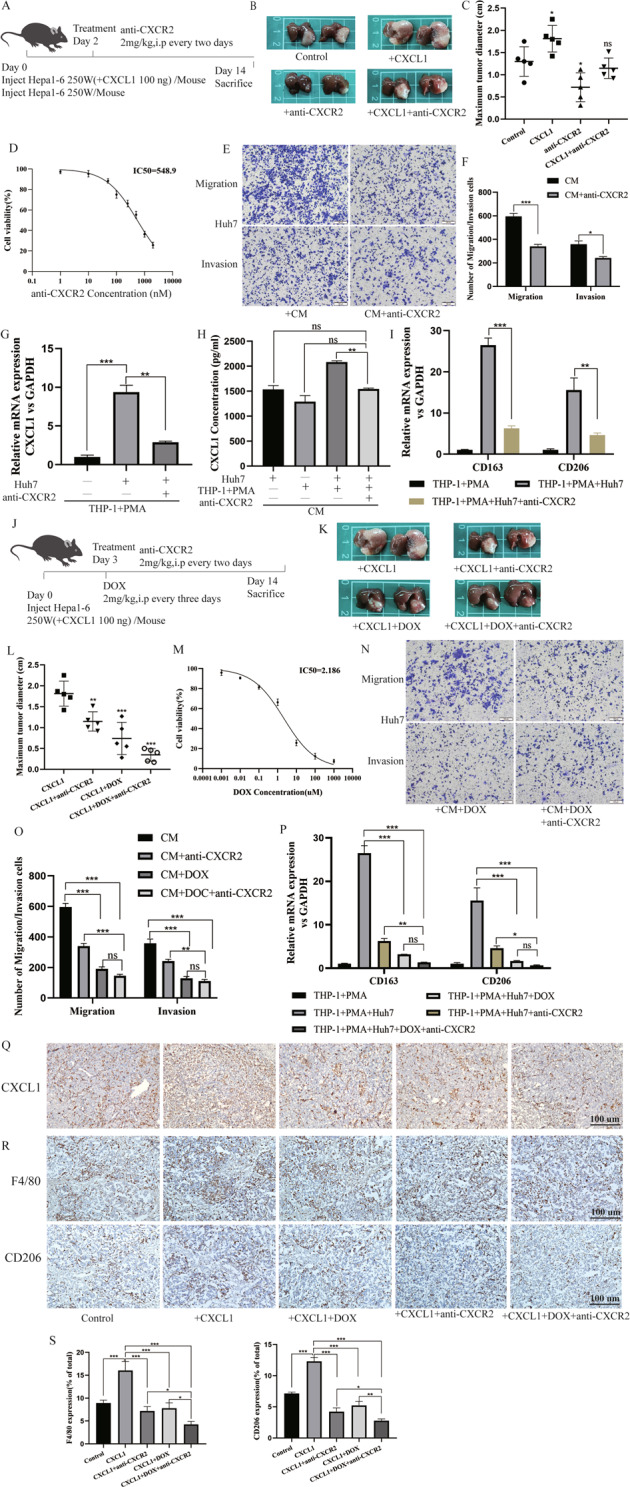
Combined treatment with DOX and anti-CXCR2 affected CXCL1 expression, tumour progression, and macrophage enrichment. **A**–**C** Photographs and maximun tumour diameter measurements of mice, which were treated with anti-CXCR2 (SB225002) antibody from two days after injecting different Hepa1–6 cells are shown, *n* = 5. **D**, **M** The IC50 values of anti-CXCR2 and DOX were measured by MTT. Data are expressed as the mean ± SEM. (*n* = 5/group). **E**, **F** Photograph and quantification of HCC cell migration and invasion in the coculture system with anti-CXCR2 antibody. **G** qRT-PCR analyses detecting the expression level of CXCL1 in macrophages after coculture and treatment with anti-CXCR2 antibody. Data are expressed as the mean ± SEM. **P < 0.01, ***P < 0.001 (*n* = 3/group). **H** CXCL1 levels in coculture supernatant (CM) and treated with anti-CXCR2 were measured by ELISA. Data are expressed as the mean ± SEM. **P < 0.01, ns, nonsignificant (*n* = 5/group). **I**, **P** qRT-PCR analyses detecting the expression levels of CD163 and CD206 in macrophages after coculture and treatment with DOX and anti-CXCR2 separately or in combination. Data are expressed as the mean ± SEM. *P < 0.05, **P < 0.01, ***P < 0.001, ns, nonsignificant (*n* = 3/group). **J**–**L** Photographs and maximun tumour diameter measurements of mice, which were treated with DOX or combined with anti-CXCR2 (SB225002) antibody from three days after injecting different Hepa1–6 cells are shown, *n* = 5. **N**, **O** Photograph and quantification of HCC cell migration and invasion in the coculture system with DOX or combined with anti-CXCR2 antibody. **Q**–**S** IHC analysis of the expression of CXCL1, F4/80, and CD206 in mouse tumour tissues that received different treatments (**Q**, **R**), and the relative expression level of F4/80 or CD206 (**S**), Scale bar, 100 μm.

To verify the effect of different treatments on tumour progression, an animal model was established by injecting mice with HCC cells or HCC cells stimulated by CXCL1 protein. Then, we treated mice with anti-CXCR2 antibody (Fig. 4A). The analysis of tumour volumes showed that anti-CXCR2 antibody inhibited tumour growth (Fig. 4B, C). To further confirm the effect of different treatments on tumour proliferation, migration, and invasion behaviour, we performed MTT and Transwell assays in vitro. The IC50 values of anti-CXCR2 antibody were calculated by MTT assays (Fig. 4D). With anti-CXCR2 antibody treatment, the number of HCC cells that metastasized through the chamber membrane was reduced (Fig. 4E, F). After collecting TAMs and CM of cells treated with anti-CXCR2 antibody, we found that the expression of CXCL1 decreased in both sample types (Fig. 4G, H).

### Combined treatment with DOX and anti-CXCR2 inhibits the proliferation and metastasis of HCC cells

Analysis of tumour volumes revealed that DOX enhanced the inhibitory effect of anti-CXCR2 antibody on tumour growth (Fig. 4J–L). According to the IC50 of DOX, we performed subsequent assays in vitro. It was clear that the combination of DOX and anti-CXCR2 antibody induced a synergistic inhibitory effect on HCC cells metastasis through the chamber membrane, indicated by suppression of both migration and invasion (Fig. 4M–O).

### Combined treatment with DOX and anti-CXCR2 inhibited M2 macrophages polarizations in vitro and reduced the enrichment of macrophages in vivo

In the coculture system, we collected TAMs to detect their phenotype by PCR. The data revealed that the expression of CD163 and CD206 was decreased after treatment with DOX or anti-CXCR2 antibody, and combined treatment with DOX and anti-CXCR2 antibody strongly inhibited the phenotype (Fig. 4I, P). In mice tumours, we found a similar result. The level of CXCL1 was higher in tumours that were injected with HCC cells stimulated with CXCL1 recombinant protein than in those injected with control cells. In contrast, treatment with DOX and anti-CXCR2 antibody individually or in combination decreased the expression of CXCL1 to different degrees (Fig. 4Q). The levels of macrophages infiltration, F4/80 expression, and CD206 expression were positively correlated with the level of CXCL1 expression (Fig. 4R, S). We hypothesize that DOX and anti-CXCR2 antibody can affect the enrichment of macrophages, especially M2-like macrophages, in the TME and can thus affect tumour progression.

### EMT of HCC cells and M2 macrophages via the pNF-κB/IL-1*β* signalling pathway to adjust the secretion of CXCL1

**Fig. 5:**
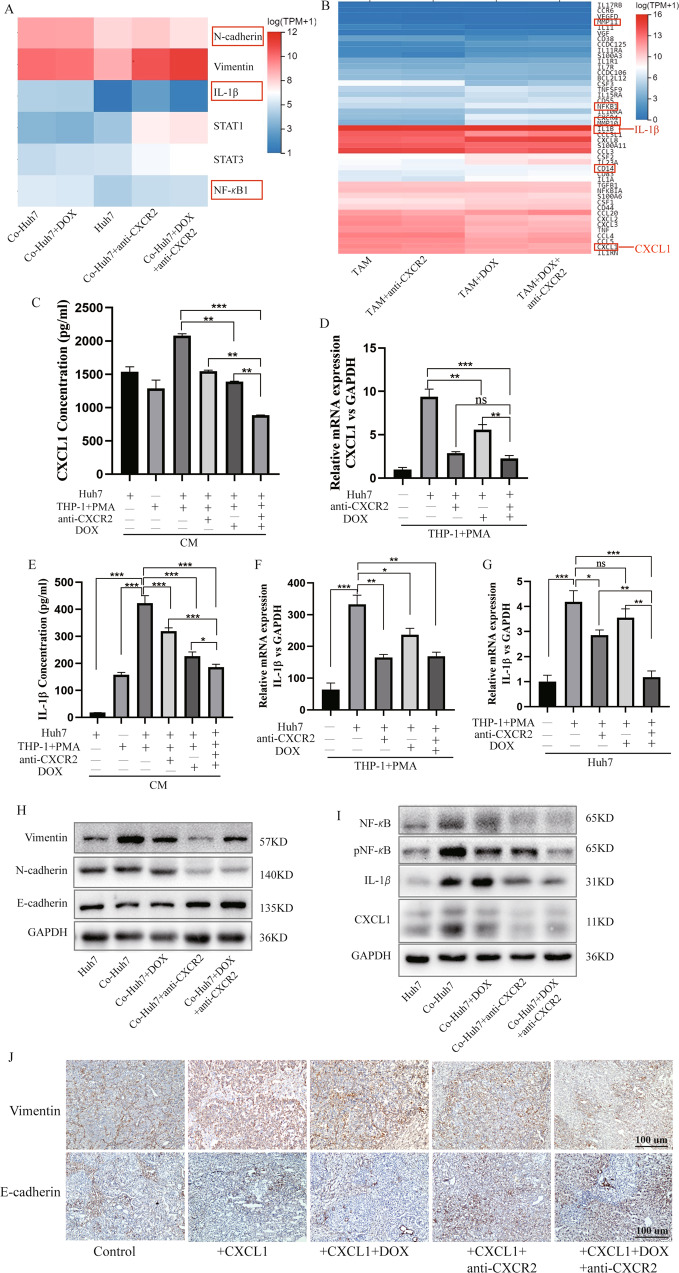
The pNF-κB/IL-1β signalling pathway is critical for CXCL1 secretion. **A**, **B** RNA sequencing of Huh7 cells. TAMs were treated with DOX or anti-CXCR2 separately or in combination to detect significant genes. **C** CXCL1 levels in the coculture supernatant (CM) of cells treated with DOX or anti-CXCR2 antibody separately or in combination were measured by ELISA. Data are expressed as the mean ± SEM. **P < 0.01, ***P < 0.001 (*n* = 5/group). **D** qRT-PCR analyses detecting the expression level of CXCL1 in macrophages after coculture and treatment with DOX or anti-CXCR2 separately or in combination. **E**–**G** The levels of IL-1*β* in CM, TAMs, and Huh7 cells were measured by ELISA and qRT-PCR. **E** Data are expressed as the mean ± SEM. *P < 0.05, ***P < 0.001 (*n* = 5/group). **D**, **F**, **G** Data are expressed as the mean ± SEM. *P < 0.05, **P < 0.01, ***P < 0.001, ns, nonsignificant (*n* = 3/group). **H** Western blot analysis of the levels of an epithelial marker (E-cadherin) and mesenchymal markers (N-cadherin and Vimentin) in Huh7 cells cocultured with DOX or anti-CXCR2 separately or in combination. **I** Western blot analysis of the expression of NF-κB, pNF-κB, IL-1*β*, and CXCL1 in Huh7 cells cocultured with DOX or anti-CXCR2 separately or in combination. **J** IHC analysis of the expression of Vimentin and E-cadherin in mouse tumour tissues that received different treatments. Scale bar, 100 μm.

In our study, CXCL1 induced macrophages to differentiate into M2-like macrophages to promote the proliferation, and EMT of HCC cells; otherwise, blocking the CXCL1-CXCR2 axis with anti-CXCR2 antibody inhibited this effect by decreasing CXCL1 expression. Combined treatment with DOX and anti-CXCR2 antibody inhibited proliferation. To determine whether the treatment can influence CXCL1 expression, CXCL1 in TAMs and in CM was detected with PCR and ELISA assays. DOX combined with anti-CXCR2 antibody further decreased the secretion of CXCL1 (Fig. 5C, D).

To identify which genes were involved in these changes in our coculture system, we collected TAMs and Huh7 cells with various treatments for analysis by RNA sequencing (RNA-seq) (Fig. 5A, B). NF-κB, IL-1*β*, and CXCL1 levels were significantly different between groups. ELISA of the coculture system showed that the expression of IL-1*β* was increased in TAMs and CM but also in Huh7 cells upon treatment. The expression of IL-1*β* was decreased to varying degrees with different treatments and was positively correlated with the expression of CXCL1 (Fig. 5C–G). Finally, we found that DOX alone did not inhibit the expression of EMT-related proteins in HCC cells. This may have some connection with resistance to DOX. However, the addition of anti-CXCR2 antibody reversed this phenomenon, the expression of N-cadherin and Vimentin was suppressed, and E-cadherin was upregulated (Fig. 5H). We observed the same result by IHC of mouse tumours (Fig. 5J). In addition, activation of the NF-κB/pNF-κB/IL-1*β*/CXCL1 pathway was clearly blocked by combined treatment with DOX and anti-CXCR2 antibody (Fig. 5I). Therefore, in this study, the results suggest that combined treatment with DOX and anti-CXCR2 antibody can block the pNF-κB/IL-1*β* signalling pathway to alter the secretion of CXCL1 and influence EMT in HCC cells and M2 macrophages.

## Discussion

In HCC patients, chronic hepatitis background is common and leads to the malignant progression of liver cancer and limits the effects of systemic treatment [7, 14]. Acting as a key effector and cytokine manufacturer, macrophages play a key role in regulating liver fibrosis, cirrhosis, carcinogenesis, EMT and metastasis [13]. Here, we found that CXCL1, a common chronic hepatitis-related cytokine, exerts protumour effects in HCC by affecting macrophages chemotaxis through the CXCL1-CXCR2 loop.

Previous studies have shown that CXCL1 is a proinflammatory cytokine in various viral and nonviral hepatitis types and is positively correlated with chronic hepatocyte injury and the progression of cirrhosis [15, 16]. The pro-tumour inflammatory effects of CXCL1 in the liver have been systematically explored in secondary liver metastatic cancer. The potential mechanisms include recruiting myeloid-derived suppressor cells (MDSCs) [12] and neutrophil infiltration to build a premetastatic niche in the liver. How elevated CXCL1 expression levels affect chronic hepatitis function in HCC has been poorly delineated. This work presented the following main findings. First, the expression of CXCL1 in liver tissue was found to be positively associated with cirrhosis, and the levels of CXCL1 expression in tumours were similar between HCC tissue and adjacent nontumor liver tissue. As a widely expressed cytokine, CXCL1 is a key marker of inflammatory response activation. In HCV, HBV, alcohol-related hepatitis and nonalcoholic steatohepatitis, CXCL1 expression in the liver background closely reflected the hepatocyte damage level and increased portal vein pressure by promoting sinusoid microthrombi [17]. It also regulates liver fibrogenesis by activating hepatic stellate cells. CXCL1 exerts its function in liver disease mainly by regulating neutrophils, Kupffer cells and myeloid-derived suppressor cells (MDSCs), and its specific receptor CXCR2 is highly enriched [18, 19]. In this research, based on evidence from HCC clinical samples, CXCL1 expression is positively correlated with the infiltration of macrophages and the ratio of CD206-positive macrophages.

Second, CXCL1 in the TME promotes HCC progression. An increase in the proportion of CD206 cells (M2 type) in tumours had a negative effect on survival in HCC patients [20]. As observed in different tumours, CXCL1 promotes tumour cell growth and EMT [5]. Here, we also found that in the HCC microenvironment, CXCL1 recruited macrophages; these macrophages were remodelled to the protumor type and increased CXCL1 secretion. This positive loop of the CXCL1-CXCR2 pathway in the TME reflects an increase in inflammation levels and the enhancement of HCC cell growth and invasion activity [9, 21]. High CXCL1 expression in the TME promotes macrophage migration, which shapes the protumor TME. The transcription of CXCL1 mainly depends on the bioactivation of NF-κB and IL-1*β*. In the HCC TME, abundant IL-1 is released by HCC and predominantly by TAMs [12, 22]. Hypoxia and necrosis of HCC cells stimulate TAMs to release abundant IL-1*β* [23]. Both IL-1*β* and CXCL1 were upregulated in coculture medium and macrophages, which coincides with the results of a previous study. The activation of IL-1*β* and phosphorylation of NF-κB, key aspects of CXCL1 activation, were confirmed in HCC cells and tissues. Administering targeted therapy agents based on molecular pathological characteristics provides promising efficacy in malignant tumour treatment, but the TME is the main challenge in overcoming drug resistance [5]. Local treatment with chemotherapy was shown to be an efficient tool to destroy the barrier. Dox is a common agent used in combination strategies to remodel the TME. Here, we found that compared to single-agent treatment, treatment with DOX followed by a CXCR2 antagonist synergistically reduced the concentration of CXCL1 and IL-1*β* in the TME. Tumour growth and EMT protein expression were significantly suppressed in the combined therapy group. According to tumour IHC staining, the proportion of CD68-positive cells was markedly decreased upon combination treatment. This showed that the combination strategy was effective in reducing peripheral macrophage recruitment into the TME and suppressing the pro-tumour phenotype induced by reducing CXCL1 expression [24]. The synergistic antitumour effects was also confirmed based on the suppression of IL-1*β* activation [18] and NF-κB phosphorylation in tumours [25].

In this study, CXCL1, a common chemokine correlated with liver inflammation and cirrhosis, was explored for its protumor function related to macrophages in HCC. CXCL1 recruits macrophages into the TME, where they are remodelled into tumour-associated macrophages and further increase the inflammation level and CXCL1 expression. The high inflammation level in the TME promotes tumour progression, partly through the CXCL1 signalling pathway. Targeting the specific receptor CXCR2 is a valid treatment for HCC. Combination of DOX with CXCR2 blockade could be a promising strategy to overcome TME barriers and enhance the efficacy of these individual drugs.

## Materials and methods

### Reagents

We purchased all the general reagents from Beyotime Biotechnology (Shanghai, China). A BCA kit, penicillin/streptomycin, MTT reagent, 4% paraformaldehyde, crystal violet and so on were used. We purchased the SYBR green real-time PCR assay kit from EnzyArtisan (Shanghai, China). RPMI1640, DMEM, 0.25% pancreatic enzymes, and FBS were purchased from Gibco, USA. Murine recombinant CXCL1 (C600168) and human recombinant CXCL1 (C600064) were obtained from Sangon (Shanghai, China). Doxorubicin (E2516), SB225002 (S7651, a potent selective CXCR2 antagonist) and phorbol 12-myristate 13-acetate (PMA) (S7791) are products of Selleck. An epithelial-mesenchymal transition (EMT) antibody kit (9782), and NF-кB p65 (8242) and phospho-NF-кB p65 (Ser536) (pNF-кB, 3033) primary antibodies were purchased from Cell Signaling Technology (Danvers, MA). Anti-IL1 beta (AF5103), anti-GAPDH (T0004), and anti-CXCL1 (AF5403) antibodies were purchased from Affbiotech. Antibodies against CD68 (ab303565), CD206 (ab64693), and F4/80 (ab300421) were obtained from Abcam. The secondary antibody was purchased from ZSBIO Company (Beijing, China).

### Cell culture and mice

Human hepatoma cell lines (Huh7), mouse hepatoma cell lines (Hepa1–6), mouse macrophages (Raw264.7) and human monocytes (THP-1) were obtained from the cell bank of the Chinese Academy of Sciences (Shanghai Academy of Biological Sciences, China). Stable CXCL1 downregulated hepatoma cells (Huh7) were obtained as previously described [15]. All cells were cultured in DMEM and RPMI 1640 supplemented with 10% FBS, 1% penicillin, and 1% streptomycin. THP-1 cells were induced to differentiate into macrophages as described by 200 ng/ml phorbol 12-myristate 13-acetate (PMA) [26]. Macrophages and HCC cells were cocultured in coculture dishes (Labselect, 14102) at a ratio of 1:1 for 48 h. According to the protocols, we used an incubator at 37 °C in a humidified atmosphere of 5% CO2 to culture all cell lines.

Male C57BL/6 J mice (4 weeks of age, 15–20 g) were obtained from Gempharmatech, Co, Ltd. The mice were maintained under specific pathogen free conditions and had free access to sterilized food and autoclaved water. All mice were randomly assigned to different group, with 5 mice in each group. These experimental procedures were approved by the Animal Ethics Committee.

Mice were injected with 0.1 ml of Hepa1–6 cell suspension (2.5 × 10^5^ cells) using a 21­gauge needle in the inferior margin of the left lobe of the liver. Anti-CXCR2 was used once every two days, while DOX was used once every three days (both 2 mg/kg, i.p.); these drugs were injected separately or in combination 2 days after cell inoculation. The control group was treated with saline. Mice were observed every day, after two weeks, and their livers were excised. A portion of the tumour tissue was fixed in 4% formalin for subsequent histologic examination, and the remaining tissue was snap frozen in liquid nitrogen and stored at −80 °C.

### Database analysis of prognostic value

The correlation between CXCL1 expression and HCC patient survival in HCC patients was analysed by GEPIA (http://gepia.cancer-pku.cn/index.html) [27]. The outcomes included overall survival (OS) and disease-free survival (DFS).

### Patient specimens and tissue microarrays

Fifty-one patients with liver cancer who underwent hepatectomy at The Second Hospital of Anhui Medical University (between 2010 and 2016) without antitumour treatment prior to surgery were enrolled. We collected their tumour tissues and paired adjacent tissues. Most of these patients have a history of hepatitis and cirrhosis. The diagnosis was confirmed by pathology. The participants involved in the study signed the informed consent. The research protocol was approved by The Second Hospital of Anhui Medical University Institutional Review Committee. Tissue microarrays (TMAs) of HCC samples from these patients were prepared (Ai-We-Er company, Wuhan, China). Fifty-one pairs of tissue were carried in TMA blocks and the sections were 4 µm [28].

### Immunohistochemistry

The mouse primary tumour tissues were harvested, and 3 µm-thick fresh frozen sections were prepared. For TMAs and frozen mouse sections, staining was performed as previously described [14, 28]. Prediluted mouse anti-CXCL1 antibody, rabbit anti-CD68, rabbit anti-CD206, E-cadherin, N-cadherin, and Vimentin were incubated overnight at 4 °C. The secondary antibody was incubated for 1 h at room temperature. After the sample was sealed neutral resin, the brown precipitates with a positive immune reaction were observed with an Olympus microscope. Two pathologists assessed the staining results. The results were further analysed with ImageJ to quantify the staining intensity.

### Quantitative real-time PCR

Total RNA was extracted from cells according to the manufacturer’s instructions. Real-time polymerase chain reaction was used to quantify the levels of CXCL1, CD68, CD206, CD163, CD14, IL-1*β*, and EMT-associated markers humans or mice. The primers used for this study are shown in Supplemental Table 1. 2^−∆∆CT^ was used to analyse the data, and the expression level of the target gene was normalized to the expression level of the reference gene GAPDH.

### Western blotting

Whole-cell protein was prepared with RIPA lysis buffer containing proteinase inhibitor and phosphatase inhibitor. Western blotting was performed according to the previous description. Protein lysates were obtained from tissues and cells. Western blotting was carried out according to a previous description [28]. The membranes were incubated at 4 °C overnight with E-cadherin, N-cadherin, Vimentin, CXCL1, IL-1*β*, NF-κB, pNF-κB, and GAPDH antibodies. Horseradish peroxidase-conjugated secondary antibodies specific for rabbit or mouse IgG were used for bind primary antibodies for protein band detection.

### MTT assay

HCC cells were inoculated into 96-well plates with 2500 cells in each well. After treatment, 6 wells were used for each group, and 10 ul of 5 mg/ml MTT solution was added. After incubation at 37 °C for 4 h, 150 μl of isopropyl alcohol was added and shaken at room temperature for 10 min, and the absorbance (OD) value was measured at 570 nm to 630 nm by a microplate reader. The half-maximal inhibitory concentration (IC50) of different drugs was calculated according to cell viability.

### Transwell assay

The migration experiment was carried out directly in a chamber (8 μm), and the invasion experiment was carried out in a chamber coated with Matrigel (Corning, USA). The cells used to evaluate migration and invasion were inoculated into the upper chamber of the chamber and cultured with serum-free medium. Medium containing 30% serum was added to the lower chamber. The cells passing through the chamber were fixed with 4% paraformaldehyde and then stained with 0.1% crystal violet. The experimental results were observed and quantified under an Olympus inverted microscope.

### ELISA

The Human GROα/CXCL1(Growth Regulated Oncogene Alpha) ELISA Kit (E-EL-H0045c, Elabscience) and human IL-1 ELISA Kit (E-EL-H0149c, Elabscience) were used according to the manufacturer’s instructions to detect the concentration of cell-free supernatants. We called the cell-free supernatants conditioned culture medium (CM). The cells were collected from the following groups: Huh7 cells were cultured separately, macrophages were cultured separately, and Huh7 cells were cocultured with macrophages and then subjected to different treatments. Measurement was performed by setting the absorbance wavelength to 450 nm.

### RNA sequencing (RNA-seq) and signal pathway enrichment analysis

Total RNA was isolated from Huh7 cells and macrophages from different groups. After that, the RNA was quantified and prepared for RNA-seq. RNA-seq datasets were processed using construction of the DNBSEQ eukaryotic chain-specific transcriptome library, as implemented through the Project (https://github.com/BGI-flexlab/SOAPnuke). According to the results of differential gene detection, hierarchical clustering analysis was carried out by using the r-package pheatmap to combine the differential genes. GO terms and KEGG pathways with corrected *p*-values < 0.05 were considered significantly enriched.

### Statistical analysis

All continuous variables were recorded as the mean ± standard deviation. Experiments were performed at least in triplicate. SPSS 22.0 and GraphPad Prism (9.0) were used for completing statistical analysis. Differences between two groups were assessed with the two-tailed Student’s unpaired *t* test, and the one-way Anova with Tukey’s multiple comparison test was used to compare differences between multiple groups. No statistical methods were used to predetermine the sample size. *P*-value < 0.05 was considered to indicate statistical significance.

**Fig. 6:**
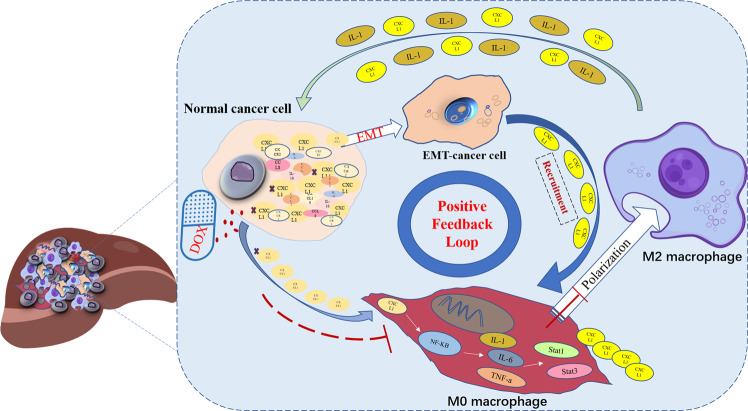
A schematic model depicting the IL-1β/CXCL1/EMT signalling loop mediates the crosstalk between HCC cells and TAMs in an inflammatory microenvironment. When exposed to inflammatory microenvironment, promoting EMT-programming in tumour cell, and up-regulates CXCL1 secretion. Moreover, M0 macrophage caused by recruitmenting more CXCL1 can be “re-educated” into M2 macrophage, and play pro-tumour effects. Accumulation of M2 macrophages IL-1*β*/CXCL1 deepens the inflammatory signalling in the TME and enhances EMT in cancer cells through the NF-κB/IL-1*β*/CXCL1 axis, which all can be inhibited by DOX or anti-CXCR2 antibody in a inflammatory microenvironment.

## Supplementary information

supplementary files

## Data Availability

Source data for Figs. 1–6 and original data of WB have been provided as source data files. All other supporting the findings of this study are available from the corresponding author upon reasonable request.
