# Supplementary material for: Blocking the CXCL1-CXCR2 axis enhances the effects of doxorubicin in HCC by remodelling the tumour microenvironment via the NF-κB/IL-1β/CXCL1 signalling pathway
Source: Cell Death Discov. 2023 Apr 10;9:120. doi: 10.1038/s41420-023-01424-y (PMC10085981; doi:10.1038/s41420-023-01424-y)
Supplement: Supplementary file 1 — supplementary files [file 41420_2023_1424_MOESM1_ESM.docx]

| **Supplemental Table 1** | |
| --- | --- |
| **Name of primer** | **Primer sequence** |
| CXCL1 | F:5-TCATAGCCACACTCAAGAAT-3  R:5-GTTGGATTTGTCACTGTTCA3-3 |
| CD68 | F:5-CTTCTCTCATTCCCCTATGGACA-3  R:5-GAAGGACACATTGTACTCCACC-3 |
| CD206 | F:5-TTCGGACACCCATCGGAATTT-3  R:5-CACAAGCGCTGCGTGGAT-3 |
| CD163 | F:5-GAAGACAGAGACAGCGGCTT-3  R:5-GGTATCTTAAAGGCTCACTGGGT-3 |
| CD14 | F:5-AAGCACTTCCAGAGCCTGTC-3  R:5-TCGTCCAGCTCACAAGGTTC-3 |
| IL-1β | F:5-CCACAGACCTTCCAGGAGAATG-3  R:5-GTGCAGTTCAGTGATCGTACAGG-3 |
| N-cadherin | F:5-CTCCAGAGTTTACTGCCATGAC-3  R:5-GTAGGATCTCCGCCACTGATTC-3 |
| E-cadherin | F:5-CATCGCTTACACCATCCTCAG-3  R:5-ACTCCTGTGTTCCTGTTAATGG-3 |
| Vimentin | F:5-GGACCTCTACGAGGAGGAGAT-3  R:5-GCCTCCAAGGAAGAGACTGA-3 |
| GAPDH | F:5-GTCTCCTCTGACTTCAACAGCG-3  R:5-ACCACCCTGTTGCTGTAGCCAA-3 |

**Supplemental Table 2**

| **Factors** | **CXCL1 expression** | |  |
| --- | --- | --- | --- |
|  | **Low(n=19)** | **High(n=32)** | ***P* value** |
| Sex (male/female) | 17/2 | 30/2 | 0.992 |
| Age (≤65/＞65 years) | 15/4 | 26/6 | 1.000 |
| AFP (low/high) | 10/9 | 13/19 | 0.405 |
| Cirrhosis (yes/no) | 11/8 | 28/4 | 0.039* |

**Correlation between CXCL1 expression in nontumour tissues from**

**HCC patients and corresponding clinicopathologic parameters**

* P<0.05


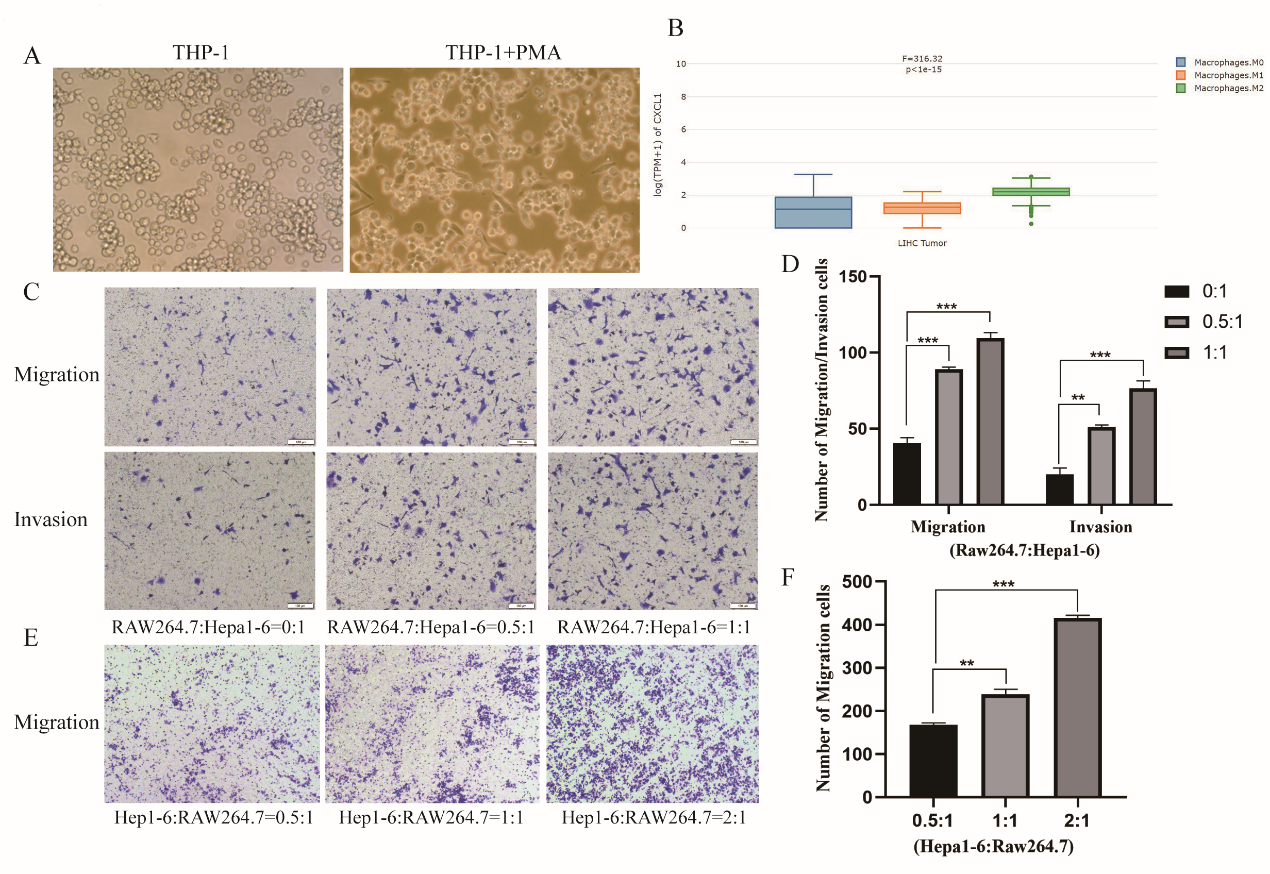


**Figure S1.** Coculture affected the metastasis of HCC cells and macrophages. (A) Images of THP-1 and PMA-primed THP-1 cells. (B) The level of CXCL1 in different types of macrophages from the GEPIA database. (C, D) Photograph and quantification of Hepa1-6 cell migration and invasion in the coculture system at different ratios of macrophages to Hepa1-6 cells. (E, F) Photograph and quantification of Raw264.7 cell migration in the coculture system at different ratios of Raw264.7 and Hepa1-6 cells.
